# Exosomal KL-6 surpasses total plasma KL-6 in the diagnosis of Interstitial Lung Disease (ILD)

**DOI:** 10.1186/s12931-026-03816-x

**Published:** 2026-07-28

**Authors:** Huimin Huang, Ruogu Lai, Junqi Li, Qian Han, Yunpeng Xiao, Xinru Wei, Jin Li, Ziyi Zhang, Yanting Fang, Yunxin Lai, Ping Zhang, Xiaoxiao Li, Jie Zhang, Shuilian Yu, Fei Yuan, Jia Li, Baoqing Sun, Jin Su

**Affiliations:** 1 Department of Clinical Laboratory, National Center for Respiratory Medicine, National Clinical Research Center for Respiratory Disease, State Key Laboratory of Respiratory Disease, Guangzhou Institute of Respiratory Health, The First Affiliated Hospital of Guangzhou Medical University, 28 Qiaozhong Road, Liwan District, Guangzhou, Guangdong 510120 China; 2 Guangzhou National Laboratory, No. 9 Xingdao Huanbei Road, Guangzhou International Bio-Island, Guangzhou, Guangdong 510005 China; 3 Medical Research Center, Tianjian Laboratory of Advanced Biomedical Sciences, The Third Affiliated Hospital of Zhengzhou University, Zhengzhou University, Zhengzhou, Henan 450052 China; 4 Feichuang Biomedical Technology (Guangzhou) Co., Ltd, Guangzhou, Guangdong 510670 China; 5 Guangzhou Jinyu Medical Laboratory Group Co., Ltd, Guangzhou, Guangdong 510005 China; 6 KingMed School of Laboratory Medicine, Guangzhou Medical University, Guangzhou, Guangdong 510180 China; 7 Department of Rheumatology, Dongguan People’s Hospital, No.78 Wandao Street, Wanjiang Road, Dongguan, Guangdong 523059 China; 8 Department of Dermatology, State Key Laboratory of Respiratory Disease, Guangdong Provincial Key Laboratory of Allergy & Clinical Immunology, The Second Affiliated Hospital, Guangzhou Medical University, Guangzhou, Guangdong 510260 China; 9 Department of Rheumatology, The Second Affiliated Hospital of Guangzhou Medical University, Guangzhou Medical University, Guangzhou, Guangdong 510010 China; 10 Department of Rheumatology and Immunology, Sun Yat-Sen Memorial Hospital, Sun Yat-Sen University, No 107 Yanjiang West Road, Guangzhou, 510120 China; 11 Department of Radiation Medicine, Faculty of Preventive Medicine, Fourth Military Medical University, No. 169 Changlexi West Road, Xincheng District, Xi’an, Shaanxi 710032 China

**Keywords:** Interstitial lung disease, Exosome extraction pre-treatment, KL-6

## Abstract

**Background:**

Krebs Von den Lungen-6 (KL-6) is an established biomarker for interstitial lung diseases (ILDs), but the utility of exosomal KL-6 versus plasma KL-6 in connective tissue disease-associated ILD (CTD-ILD) remains unclear.

**Methods:**

We integrated single-cell transcriptomic re-analysis (GSE135893) with a multi-center cohort of 223 subjects, including 167 ILD, 22 CTD without ILD[CTD (Non-ILD)], 34 healthy controls(HCs). Plasma and exosomal KL-6 were measured, and diagnostic performance was evaluated via Receiver operating characteristic(ROC) analysis. Ten progressive ILD patients were monitored longitudinally.

**Results:**

Single-cell sequencing revealed a significantly stronger correlation between Mucin 1 (MUC1)and exosomal markers (CD63/CD81) in alveolar type II cells from ILD patients compared to HCs, suggesting a potential vesicular association of KL-6. Clinical validation demonstrated that both plasma and exosomal KL-6 levels were significantly elevated in the ILD group. Crucially, for distinguishing CTD-ILD patients with negative plasma KL-6 results [CTD-ILD (plasma KL-6-)] from HCs, exosomal KL-6 [Area under the curve(AUC) = 0.900] outperformed plasma KL-6 (AUC = 0.879). More importantly, in differentiating CTD-ILD (plasma KL-6-) from CTD (Non-ILD), exosomal KL-6 again showed superior diagnostic accuracy (AUC = 0.782, sensitivity = 90.0%, specificity = 77.3%) compared to plasma KL-6 (AUC = 0.705, sensitivity = 65.0%, specificity = 81.8%). Plasma and exosomal KL-6 concentrations were highly correlated (rs = 0.968, *p* < 0.001). In longitudinal monitoring, both biomarkers increased in patients with radiographic disease progression.

**Conclusions:**

Exosomal KL-6 is superior to plasma KL-6 for diagnosing CTD-ILD, especially in plasma KL-6-negative patients, and shows potential for monitoring disease progression.

**Supplementary Information:**

The online version contains supplementary material available at https://doi.org/10.1186/s12931-026-03816-x.

## What is already known about this subject?

KL-6 is an established serum biomarker for ILD. However, approximately 15% of patients with radiologically confirmed ILD test negative for plasma KL-6, limiting its diagnostic sensitivity in early or atypical cases. Exosomes hold potential as carriers of disease-specific biomarkers, but the presence and clinical utility of exosomal KL-6 in ILD, particularly in CTD-ILD, remained unclear .

## What does this study add?

This study is the first to integrate single-cell transcriptomic analysis with a multi-center clinical cohort, demonstrating that exosomal KL-6 outperforms plasma KL-6 in diagnosing CTD-ILD, especially in patients who are plasma KL-6 negative. Exosomal KL-6 showed higher diagnostic accuracy (AUC 0.782 vs. 0.705) and sensitivity (90.0% vs. 65.0%) in differentiating CTD-ILD (plasma KL-6-) from CTD (Non-ILD), and exhibits potential for monitoring disease progression.

## How might this impact on clinical practice?

Exosomal KL-6 could serve as a more sensitive diagnostic tool, aiding in the early identification of CTD-ILD patients who are plasma KL-6 negative, enabling earlier intervention and risk stratification. Its stability and disease specificity also make it a promising candidate for longitudinal monitoring of disease activity and treatment response, guiding clinical management decisions in rheumatology and respiratory medicine.

## Introduction

ILDs are a heterogeneous group of disorders characterized by alveolar epithelial injury and variable degrees of pulmonary fibrosis, with idiopathic pulmonary fibrosis (IPF) and CTD-ILD being the most widely recognized subtypes. In the 2015 the American Thoracic Society(ATS)/ European Respiratory Society Congress (ERS) classification criteria further introduced “interstitial pneumonia with autoimmune features (IPAF)” to describe patients with autoimmune traits not fulfilling definitive CTD criteria [1]. Histologically, IPF is defined by usual interstitial pneumonia (UIP), while CTD-ILD and IPAF often exhibit non-specific interstitial pneumonia (NSIP) or mixed patterns on high-resolution computed tomography (HRCT) [2–5]. Despite advances in imaging and histopathology, early diagnosis remains challenging due to overlapping clinical features and the suboptimal sensitivity/specificity of current tools for atypical or early-stage cases.

KL-6, a fragmented epitope of mucin 1 (MUC1), is a well-established serum biomarker reflecting alveolar epithelial damage and ILD progression, although KL-6 is also elevated in adenocarcinoma [6] and has been associated with disease severity in specific lung infections such as COVID-19 [7]. KL-6/MUC1 is a giant mucin protein with a heavily glycosylated extracellular domain that extends up to 200–500 nm from the surface of alveolar type II epithelial cells (AT2) [8–10]. The split-off of KL-6 epitopes from membrane-located MUC-1 is induced by mechanical forces and proteolytic cleavage upon damage to AT2 [11–13]. Owing to its large size, the release of substantial amounts of soluble KL-6 into the bloodstream is thought to require significant disruption of the alveolar–vascular barrier, as demonstrated in berylliosis [14]. In addition, KL-6/MUC1 translocated from alveoli to blood stream was influenced by genetically determined molecular size [15]. Therefore, a high level of circulating KL-6 reflects severe damage to alveolar epithelial cells and alveolar vascular dysfunction, but a low level of circulating KL-6 may be influenced significantly by both the molecular size and the severity of alveolar vascular injury [14]. Moreover, the MUC1 ectodomain has been shown to act as a decoy receptor for flagellin during Pseudomonas aeruginosa infection [16].

Notably, approximately 15% of patients with radiologically confirmed ILD lesions present with negative KL-6 test results, leading to the need for more sensitive detection strategies [17, 18]. Exosomes—small extracellular vesicles (30–150 nm) enriched with cell-specific proteins and nucleic acids—have emerged as promising biomarker [19]. Owing to the decoy receptors effects and the potential influence of the size of MUCs, the presence and diagnostic utility of exosome isolation in KL-6 remain unconfirmed. Recently, the improvement of MUC protein detection sensitivity by exosome isolation had been applied in exosomal CA125 [20, 21]. It suggested that exosome isolation may improve KL-6 sensitivity and reveal the heterogeneity of KL-6 across ILD that is undetectable through conventional paired-antibody immunoassay methods.

To enhance the sensitivity of KL-6 detection in ILD, we applied an advanced exosome isolation technique (EXODUS) as a pre-treatment step. This study was designed to bridge fundamental biology with clinical application by integrating single-cell transcriptomic analysis, multi-cohort clinical validation, and longitudinal monitoring. It aimed to address three pivotal questions: (1) What is the cellular origin and vesicular association of KL-6, and is exosomal KL-6 a detectable and stable entity in the circulation of ILD patients? (2) Does exosomal KL-6 demonstrate superior diagnostic sensitivity and specificity compared to plasma KL-6, especially in the critical subgroup of patients with plasma KL-6 levels below the conventional diagnostic threshold? (3) Does exosomal KL-6 serve as a reliable biomarker for tracking disease progression over time?

## Methods

### Research subjects

**Fig. 1:**
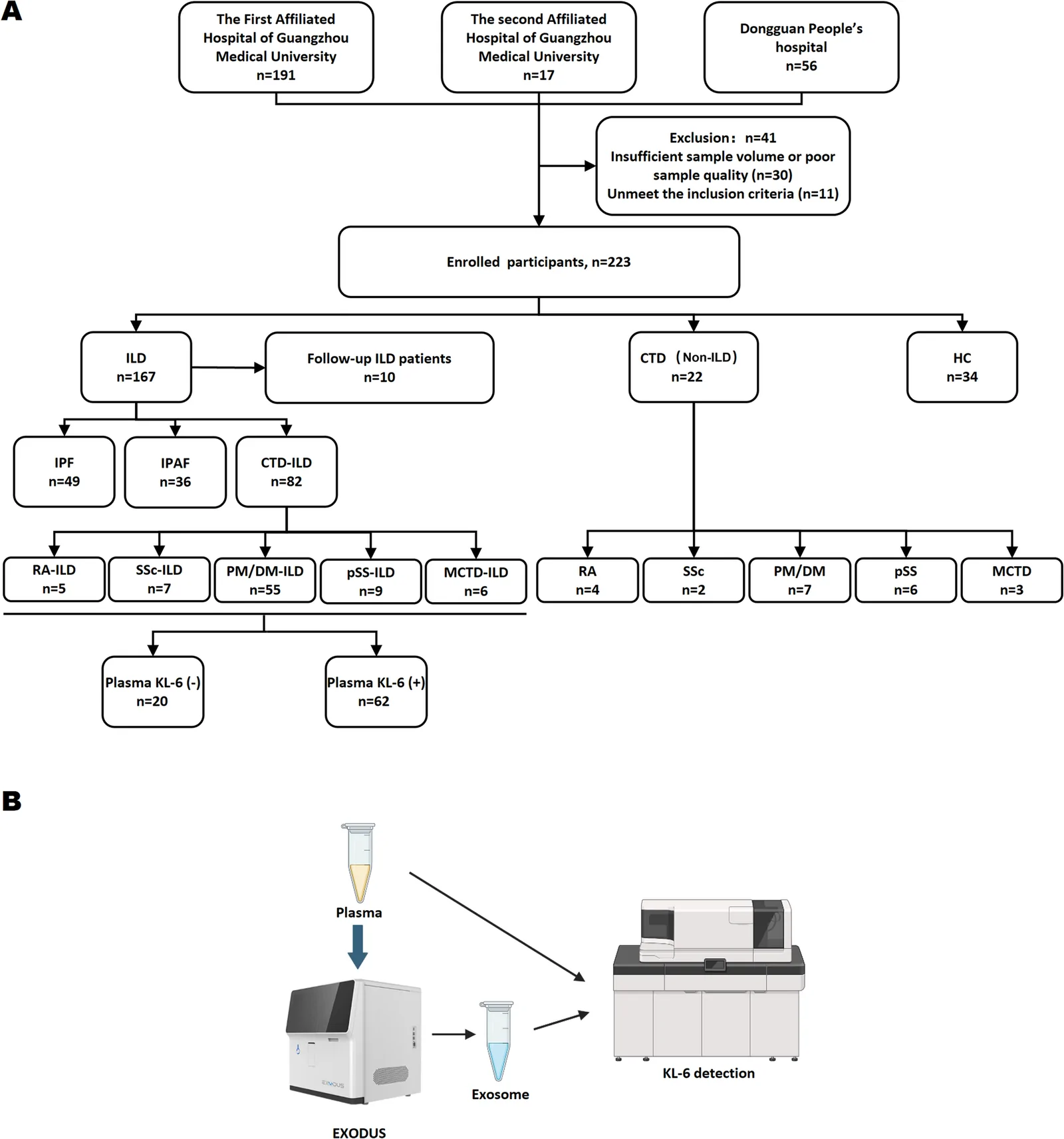
The flowchart of the study design. **A** Study framework diagram. **B** Extraction and detection of exosomal KL-6

This study included 223 subjects: 167 ILDs, 22 CTD (Non-ILD), and 34 HCs from three centers, including the First Affiliated Hospital of Guangzhou Medical University (164 enrolled, 27 excluded ), the Second Affiliated Hospital of Guangzhou Medical University(14 enrolled, 3 excluded) and the Dongguan People’s Hospital(45 enrolled, 11 excluded). The CTD-ILD group comprised 82 patients, including RA-ILD (*n* = 5), SSc-ILD (*n* = 7), PM/DM-ILD (*n* = 55), pSS-ILD (*n* = 9), and MCTD-ILD (*n* = 6). The CTD (Non-ILD) group comprised 22 patients, including RA (*n* = 4), SSc (*n* = 2), PM/DM (*n* = 7), pSS (*n* = 6), and MCTD (*n* = 3). The multidisciplinary team (MDD) composition: Diagnosis of ILD and its subtypes (IPAF, IPF, CTD-ILD) for all enrolled patients was confirmed by a fixed MDD team consisting of two senior pulmonologists, one radiologist, and one rheumatologist. This retrospective study was approved by the Ethics Committees of all participating centers. All enrolled patients were hospitalized individuals who provided written informed consent during their hospitalization, permitting the use of residual biological samples and related clinical data for medical research. The flowchart of the study design is shown in Fig. 1A.

### The case group

Inclusion criteria:

1. Age ≥18 years;
2.  ILD group: patients were diagnosed with ILD based on internationally recognized diagnostic criteria [22] , HRCT showing typical ILD imaging features, such as reticular opacities, honeycombing, or ground-glass opacities, pulmonary function tests demonstrating restrictive ventilatory dysfunction [e.g., reduced total lung capacity (TLC) or vital capacity (VC), and decreased diffusing capacity for carbon monoxide (DLCO)] and presence of ILD-related clinical symptoms, such as progressive dyspnea, dry cough, or bilateral basal crackles. This group was further stratified into the following subtypes based on established guidelines:
  IPAF: Patients meeting the 2015 ERS/ATS classification criteria for IPAF [1].
  IPF: Patients meeting the 2018 ATS/ERS/IRS/ALAT diagnostic criteria for IPF, with HRCT and/or histopathological pattern of usual interstitial pneumonia (UIP) [23].
  CTD-ILD: Patients with a confirmed diagnosis of a specific CTD according to internationally accepted classification criteria [24, 25] and concurrent ILD as defined above.
3. CTD (Non-ILD) group: Patients were required to have a confirmed diagnosis of a specific CTD according to internationally accepted classification criteria [26–30], while demonstrating no evidence of ILD through comprehensive assessment including: absence of characteristic ILD features on HRCT, no clinically significant ILD-related respiratory symptoms, and no restrictive ventilatory dysfunction or impaired diffusing capacity on pulmonary function testing when performed. This stringent criteria ensured the enrollment of a pure CTD control group without pulmonary involvement for comparative analysis.

### The HCs group

Inclusion criteria: (1) age ≥18 years ; (2) without respiratory diseases(e.g., asthma, chronic obstructive pulmonary disease, ILD, pneumonia, bronchitis); (3) normal laboratory test results: routine blood tests, liver and kidney function, and inflammatory markers (such as C-reactive protein and erythrocyte sedimentation rate) are within normal ranges; (4) no significant pulmonary imaging abnormalities: Chest X-ray or HRCT examination shows no qualitative lesions, fibrosis, or other abnormal manifestations.

#### Exclusion criteria

(1) age < 18 years; (2) patients with active pulmonary tuberculosis; (3) breastfeeding or pregnant women; (4) malignant tumors (including breast cancer, liver cancer, etc.) ; (5) cardiovascular diseases (e.g., heart failure, coronary artery disease) ; (6) liver diseases (e.g., hepatitis, cirrhosis) ; (7) renal diseases (e.g., chronic kidney disease, renal failure) .

### Sample collection & processing

Plasma was collected from peripheral blood in Ethylenediaminetetraacetic Acid (EDTA) tubes and centrifuged at 4℃ for 20 min at 2000 g; the supernatant was then centrifuged at 4℃ for another 30 min at 12,000 g. 200 µl of plasma from the middle of the obtained supernatant was added to 350 µl of phosphate buffer saline (PBS). Exosomes were isolated using the EXODUS system (Shenzhen Huixin Biomedical Technology Co., Ltd., Guangdong, China) [31]. A small EXODUS chip was used to isolate the exosomes using a modified purification procedure. After 10 min, the exosomes were isolated, resuspended in 400 µl of PBS. Plasma and exosomes were stored at -80℃ (Fig. 1B).

### KL-6 measurement

KL-6 levels were measured using the automatic chemiluminescence immunoassay system (Shine i2910) with reagents from Guangzhou Kangrun Biotechnology Co., Ltd (Guangzhou, China). The diagnostic cutoff value was set at 500 U/mL, as established in literature based on optimal balance between sensitivity for ILD detection and specificity in healthy populations [32].

### Clinical data & HRCT assessment

Collection of baseline demographics/clinical data. For longitudinal sub-study (10 ILD patients): Standardized assessment of chest HRCT progression as the reference for disease activity. All chest HRCT images were assessed by two radiologists. Radiographic progression was defined as an increase in the extent of fibrotic changes (e.g., honeycombing, traction bronchiectasis, reticulation) or the appearance of new ground‑glass opacities on follow‑up HRCT. For the 10 patients in the longitudinal study, HRCT was performed at baseline and at follow‑up, and disease progression was determined by side‑by‑side comparison of the two scans.

### Bioinformatic analysis

The single-cell dataset GSE135893 was analyzed and annotated with R 4.3.0 software(R Foundation for Statistical Computing, Vienna, Austria), and the R package Seurat V4 Satija Lab, New York, NY, USA was used to standardize and normalize the single-cell data [33]. The correlations between MUC1 and disease-related genes in various diseases [unclassifiable ILD, IPF, idiopathic nonspecific interstitial pneumonia (iNSIP), sarcoidosis, chronic hypersensitivity pneumonitis(cHP)] and the gene expression levels of ILD related membrane proteins in different cell types in multiple diseases were analyzed.

### Statistical analysis

Statistical analysis was performed using SPSS 19.0. Normally distributed data are presented as mean and standard deviation, nonparametric quantitative data are presented as median (interquartile range). Horizontal differences among multiple non-parametric groups were tested using the Kruskal-Wallis test, while differences between two groups were examined with the Mann-Whitney U test. Receiver Operating Characteristic (ROC) curve analysis, calculation of Area Under the Curve (AUC), sensitivity, specificity. Determination of optimal cutoff using Youden’s Index. Spearman’s rank correlation (rs) between plasma and exosomal KL-6. The difference in positive rates was tested by the chi-squared test. *P* < 0.05 was considered statistically significant.

## Results

### Increased correlations between MUC1 and CD63/CD81 expression in AT2 cells in ILD

**Fig. 2:**
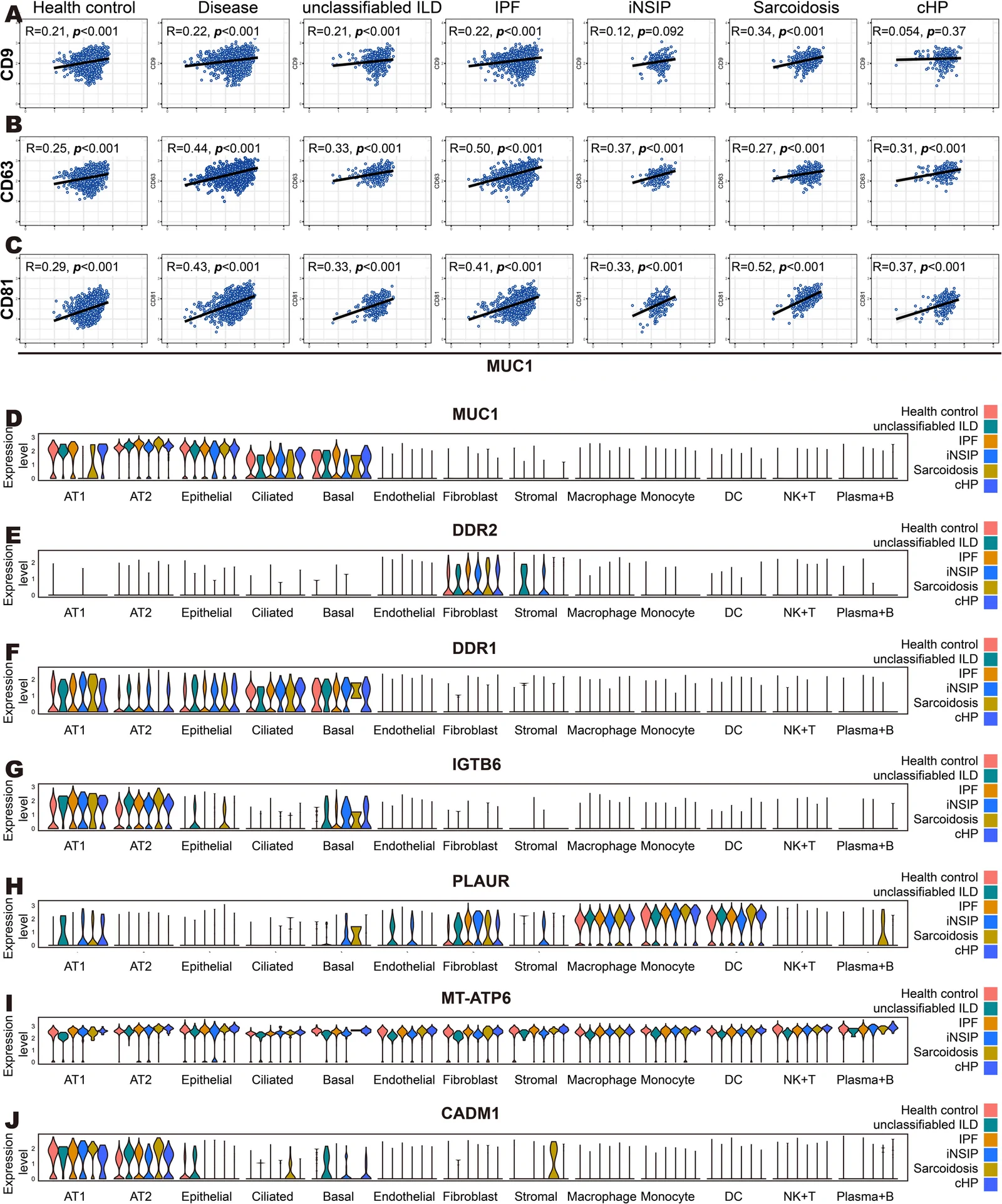
MUC1 is associated with exosomal markers and specifically upregulated in ILD within pulmonary epithelial cells. **A**–**C** The increasing correlation between MUC1 and extracellular vesicle markers in unclassified ILD, IPF, iNSIP, sarcoidosis, and cHP patients. Data were analyzed using Pearson correlation analysis. **D**–**J** The membrane biomarker (DDR2、DDR1、ITGB6、PLAUR、MT、ATP6、CADM1) expression of CTD-ILD and PPF was reanalyzed in unclassified ILD, IPF, iNSIP, sarcoidosis, and cHP patients

Whether exosomal KL-6 is present in patients with ILD has not been clarified. In this study, we reanalyzed the scRNAseq data (GSE135893) of patients with chronic ILD (including unclassified ILD, IPF, iNSIP, sarcoidosis, and cHP) and HCs [33]. Previous studie [34, 35] have provided important insights into the proteomic and biomarker landscape of ILD, which informed our analytical approach. We found that, in AT2 cells, the Pearson correlation coefficient between MUC1 and the extracellular vesicle markers CD63/CD81 was 0.25/0.29 in HCs and increased to 0.44/0.43 in ILD patients (Fig. 2A–C). KL-6 is a specific glyco-epitope on the MUC1 protein. The increased correlations between MUC1 and CD63/CD81 in AT2 cells in different ILDs indicated that KL-6 may be secreted into exosomes by AT2 cells. We further analyzed the levels of MUC1, Discoidin Domain Receptor Tyrosine Kinase 2(DDR2), Discoidin Domain Receptor Tyrosine Kinase 1(DDR1), Integrin Subunit Beta 6 (ITGB6), Plasminogen Activator, Urokinase Receptor (PLAUR), Mitochondrially Encoded ATP Synthase Membrane Subunit 6 (MT-ATP6), and Cell Adhesion Molecule 1 (CADM1), which are membrane proteins and biomarkers of CTD-ILD or progressive fibrosing interstitial lung disease (PPF). MUC1, but not other biomarkers, was elevated in both alveolar and airway epithelial cells in all chronic ILDs (Fig. 2D–J). These results suggested that damaged AT2 or airway epithelial cells may release exosomes containing the MUC1 epitope KL-6. Thus, we isolated exosomes from the plasma of patients with IPAF, IPF CTD-ILD or CTD (Non-ILD) and HCs, and detected the levels of exosomal KL-6 to determine their diagnostic potential in ILDs.

### Participant characteristics

A total of 223 subjects were enrolled: 167 patients with ILD (including 36 with IPAF, 49 with IPF, and 82 with CTD-ILD), 22 patients with CTD (Non-ILD), and 34 HCs. There was a significant difference in age among the three groups (*H* = 12.737, *P* = 0.002), with the ILD group being older. Among the ILD subtypes, the IPF group had the highest median age (65.0 years, IQR: 58.5–73.0), followed by IPAF (61.5 years, IQR: 53.5–69.0) and CTD-ILD (54.5 years, IQR: 48.8–62.5), ( *H* = 39.379, *P* < 0.001).The gender distribution also differed, with a higher proportion of females in the CTD (Non-ILD) group(77.3%). Within the ILD subtypes, IPF showed a male predominance (89.8% male), while CTD-ILD had a female predominance (85.4% female); IPAF had a relatively balanced gender distribution (58.3% female) (Table 1).

**Table 1:** Baseline characteristics of study participants

Variables	IPAF	IPF	CTD-ILD	CTD (Non-ILD)	HC	H	*P*
Patients, n	36	49	82	22	34		
Age (years)	61.5(53.5,69.0)	65.0 (58.5,73.0)	54.5 (48.8,62.5)	55.0 (46.8,60.3)	54.0 (47.8,59.0)	12.737	0.002
Sex, M/F	15/21	44/5	12/70	5/17	16/18		
Plasma KL-6 (U/mL)	913.0 (355.3,1542.5)	1137.0 (558.0,1928.0)	1114.5(510.6,1877.6)	256.4 (205.4,354.7)	209.5 (156.5,272.5)	97.614	< 0.001
Exosomal KL-6 (U/mL)	368.1 (113.5,980.4)	579.7 (276.7,1101.6)	463.0 (214.3,924.4)	82.7 (64.2,125.6)	76.5 (56.6,100.0)	98.280	< 0.001

### Correlations between plasma KL-6 and exosomal KL-6

**Fig. 3:**
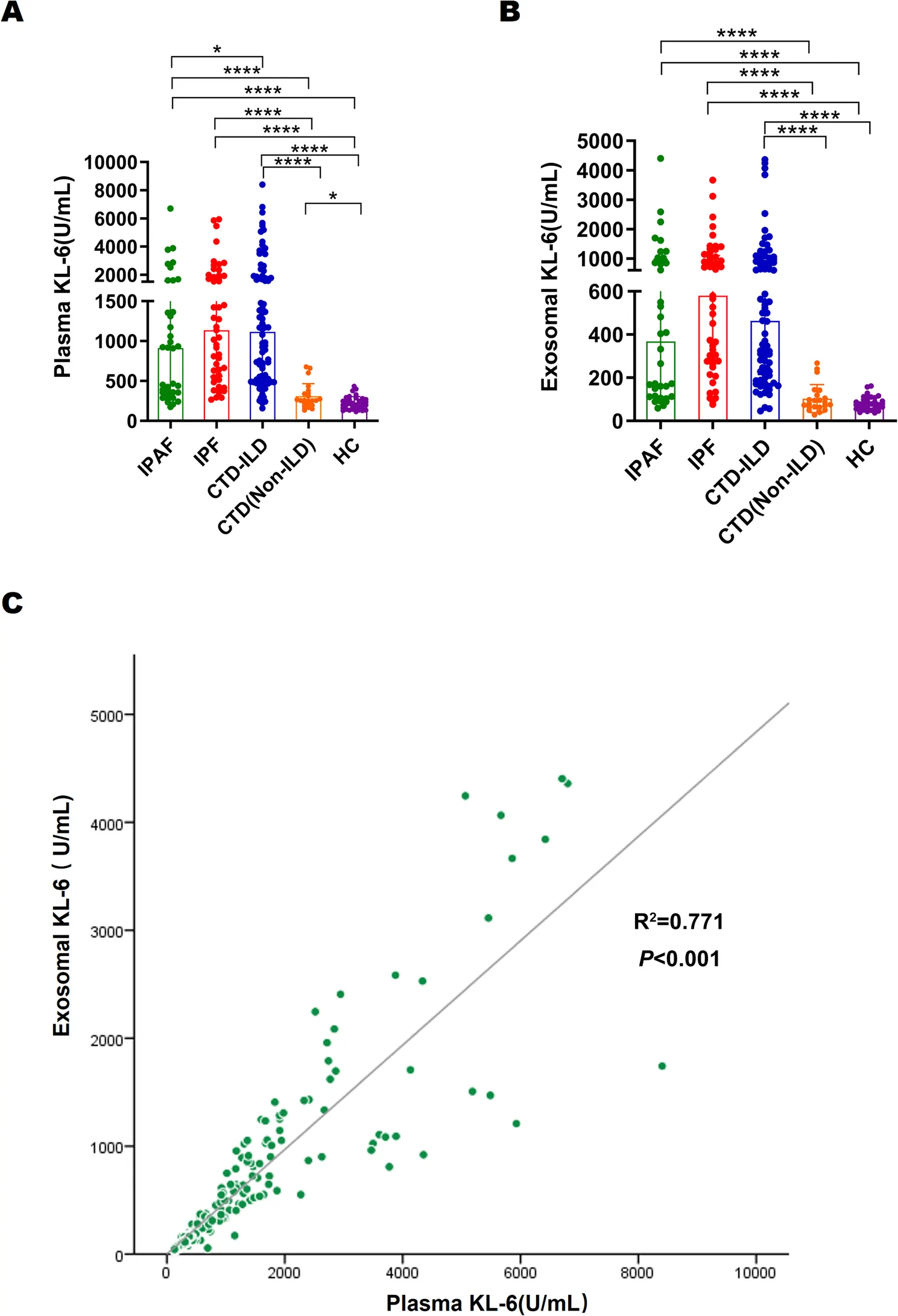
Comparison and Correlation of Plasma and Exosomal KL-6 Levels Across Different Subject Groups. **A** Comparison of plasma KL-6 levels across the five study groups. IPAF (*n* = 36), IPF (*n* = 49), CTD-ILD (*n* = 82), CTD (Non-ILD) (*n* = 22), and HC (*n* = 34). **B** Comparison of exosomal KL-6 levels across the five study groups. **C** Correlation analysis between plasma KL-6 and exosomal KL-6 levels in all 223 subjects. The Spearman rank correlation test was used

Plasma KL-6 levels were markedly elevated in the ILD group [989.0(481.0-1775.0) U/mL] compared to both the CTD (Non-ILD) group [256.4(205.4-354.7) U/mL; *Z*=-6.149, *P* < 0.001)] and HC group [209.5(156.5-272.5) U/mL; Z=-8.468, *P* < 0.001]. Among ILD subtypes, plasma KL-6 levels were highest in IPF [1137.0 (558.0-1928.0) U/mL] and CTD-ILD [1114.5 (510.6-1877.6) U/mL] with IPAF patients having lower levels [913.0 (355.3-1542.5) U/mL] (Fig. 3A). Similarly, exosomal KL-6 levels were significantly higher in ILD group [481.2 (198.8-1007.1) U/mL] than in CTD (Non-ILD) group[ 82.7 (64.2-125.6) U/mL; *Z*=-6.397, *P* < 0.001)] and HC group [76.5 (56.6–100.0) U/mL; *Z*=-8.382, *P* < 0.001)]. Within ILD subtypes, exosomal KL-6 levels were highest in IPF [579.7 (276.7-1101.6) U/mL] and CTD-ILD [463.0 (214.3-924.4) U/mL], with IPAF patients having lower levels [(368.1 (113.5-980.4) U/mL] (Fig. 3B). While exosomal KL-6 concentration was positively correlated with plasma KL-6 concentration overall (rs = 0.968, *P* < 0.001), a notable proportion of patients exhibited deviations from the regression line, with some distributed above and others below it (Fig. 3C). This scatter pattern suggests potential biological or clinical heterogeneity in the release or clearance of KL-6 between plasma and exosomal compartments.

### Exosomal KL-6 showed better diagnostic performance than plasma KL-6

**Fig. 4:**
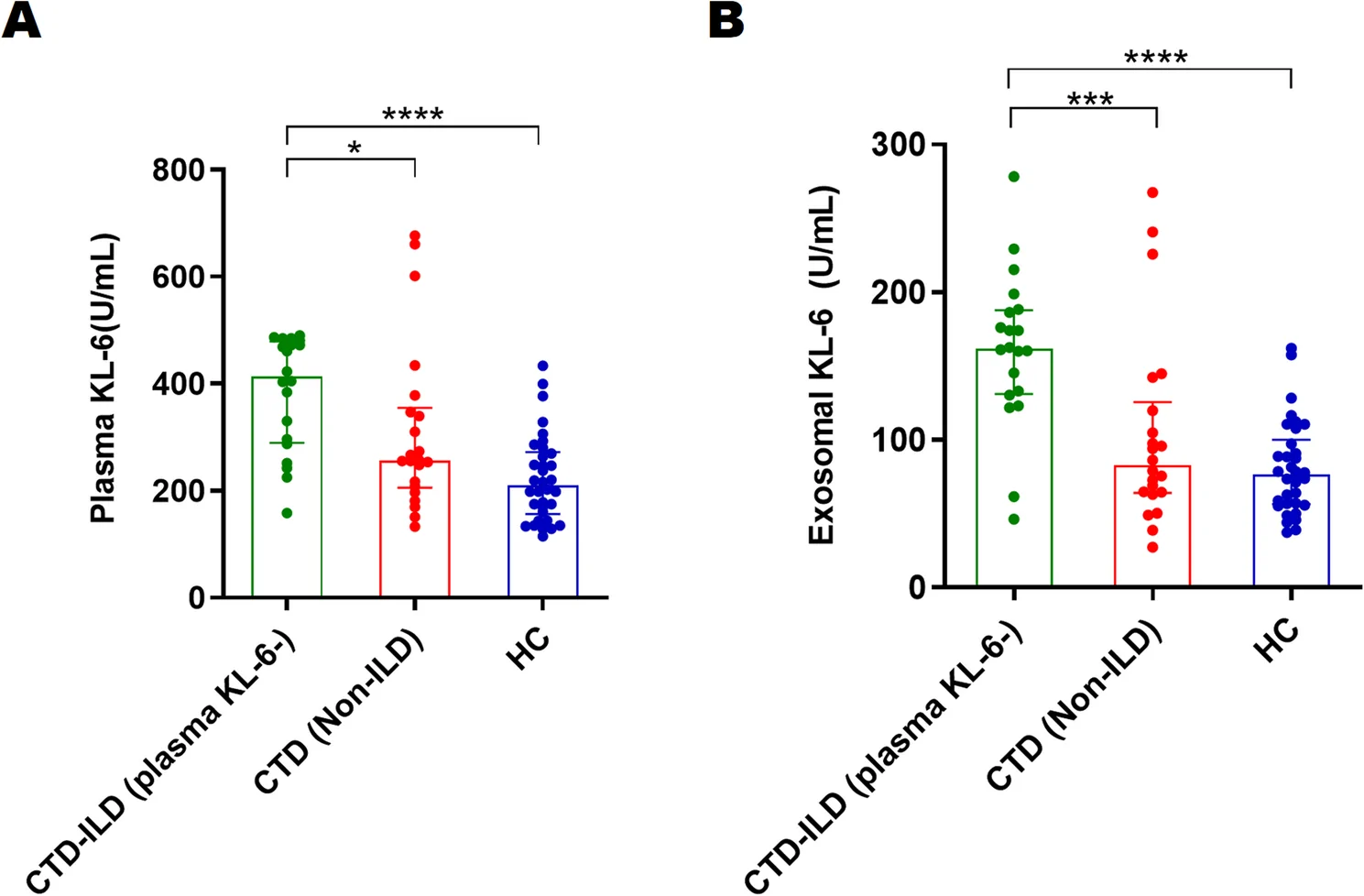
Comparison of (**A**) plasma KL-6 and (**B**) exosomal KL-6 levels among three groups: CTD-ILD (plasma KL-6-) (*n* = 20, selected by plasma KL-6 < 500 U/mL), CTD (Non-ILD) (*n* = 22), and HC (*n* = 34). Note that the CTD (Non-ILD) group includes some patients with plasma KL-6 > 500 U/mL, which is consistent with the known biology of CTD without ILD

This sub-analysis included 20 CTD-ILD (plasma KL-6-), 22 CTD (Non-ILD), and 34 HCs. Age was comparable across all three groups (*H* = 0.507, *P* = 0.776), though gender distribution varied. Crucially, significant differences were observed for two biomarkers between the groups (all *P* < 0.001). Plasma KL-6 levels were significantly elevated in the CTD-ILD (plasma KL-6-) group [413.8(290.0, 478.9) U/mL] compared to both the CTD (Non-ILD) group [256.4(205.4-354.7) U/mL; *Z*=-2.267, *P* = 0.023] and the HC group [209.5 (156.5-272.5) U/mL; *Z*=-4.621, *P* < 0.001] (Fig. 4A). In contrast, exosomal KL-6 levels were significantly higher in the CTD-ILD (plasma KL-6-) group [161.8 (133.00-176.16) U/mL] than in both the CTD (Non-ILD) group [82.7(64.2-125.6) U/mL; *Z*=-3.123, *P* = 0.002] and the HC group [76.5(56.6–100.0) U/mL; *Z*=-4.872, *P* < 0.001] (Fig. 4B).

**Fig. 5:**
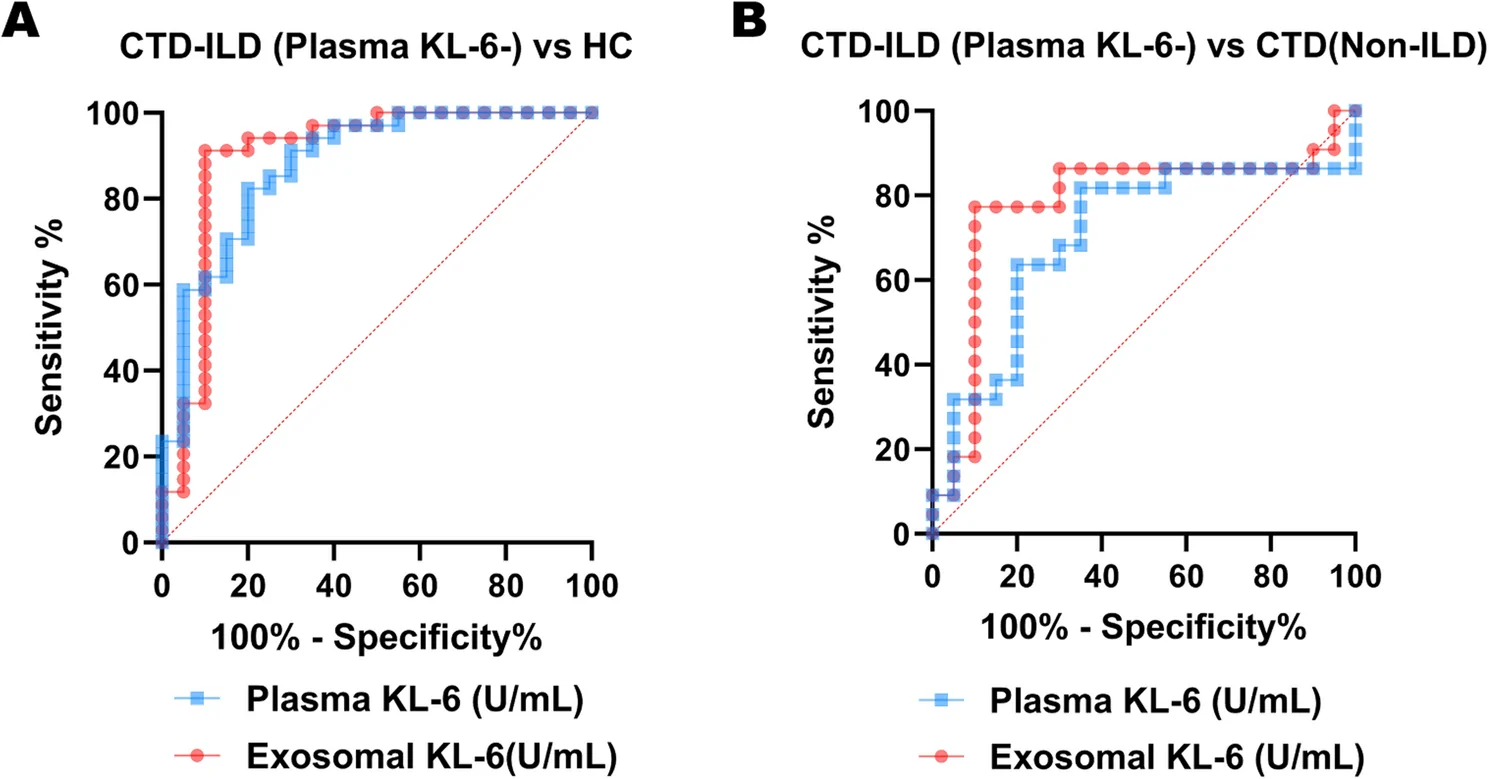
ROC curves for Exosomal KL-6 and Plasma KL-6 distinguishing (**A**) CTD-ILD (plasma KL-6-) from HCs and (**B**) CTD-ILD (plasma KL-6-) from CTD (Non-ILD)

For distinguishing CTD-ILD (plasma KL-6-) patients from HCs, exosomal KL-6 again showed the highest diagnostic efficacy among the individual markers, achieving an AUC of 0.900. At its optimal cutoff (119.125 U/mL, Youden’s index = 0.812), exosomal KL-6 provided a sensitivity of 90.0% and a specificity of 91.2%. This performance exceeded that of plasma KL-6 (AUC = 0.879, cutoff = 287.230U/mL, sensitivity = 80.0%, specificity = 82.4%). The diagnostic performance of plasma KL-6 and exosomal KL-6 for identifying CTD-ILD (plasma KL-6-) patients was evaluated using ROC curve analysis (Fig. 5A). When distinguishing CTD-ILD (plasma KL-6-) patients from CTD (Non-ILD), exosomal KL-6 demonstrated superior diagnostic accuracy with AUC of 0.782. At its optimal cutoff value (120.87 U/mL, Youden’s index = 0.673), exosomal KL-6 achieved a sensitivity of 90.0% and a specificity of 77.3%. Both the AUC and the sensitivity values for exosomal KL-6 were higher than those for plasma KL-6 (AUC = 0.705, cutoff = 381.58 U/mL, sensitivity = 65.0%, specificity = 81.8%) (Fig. 5B). These results consistently demonstrate that exosomal KL-6 outperforms plasma KL-6 in distinguishing ILD comorbidity from CTD patients.

### Plasma KL-6 and exosomal KL-6 levels rise during ILD progression

**Fig. 6:**
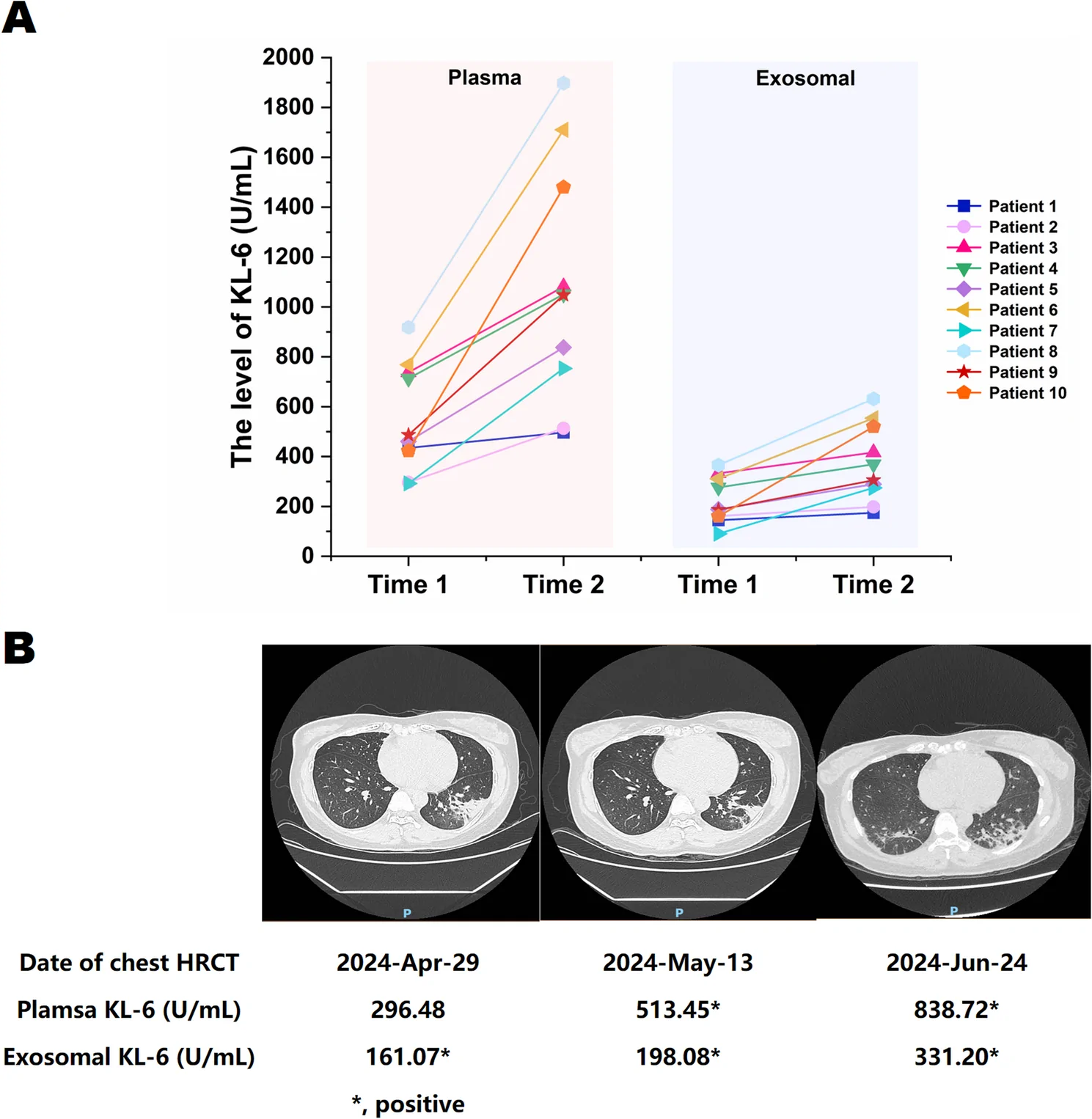
Serial changes in KL-6 levels correlated with radiographic progression in 10 CTD-ILD patients. **A** Box plots showing the longitudinal levels of Plasma KL-6 and Exosomal KL-6 in 10 patients with documented CTD-ILD progression. Both biomarkers demonstrated a significant increasing trend from Time 1 (baseline) to Time 2 (follow-up), consistent with disease activity. The individual intervals were: Patient 1: 143 days, Patient 2: 12 days, Patient 3: 70 days, Patient 4: 326 days, Patient 5: 74 days, Patient 6: 31 days, Patient 7: 98 days, Patient 8: 181 days, Patient 9: 107 days, Patient 10: 173 days. The short interval for Patient 2 (12 days) was due to an acute exacerbation requiring early re-hospitalization, while the longest interval for Patient 4 (326 days) reflected slow disease progression with a longer follow-up schedule. All HRCT examinations were performed within 3 days before or after the blood collection dates. **B** A representative case illustrating the concomitant rise in KL-6 levels with fibrotic progression on HRCT. The upper panel shows chest HRCT images at three time points, revealing a progressive fibrotic pattern. The lower panel displays the corresponding biochemical data: during this follow-up period, the exosomal KL-6 level increased from 161.07 U/mL to 331.2 U/mL, while the plasma KL-6 level rise from 296.48 U/mL to 838.72 U/mL

Plasma KL-6 and exosomal KL-6 levels showed an increasing trend in patients with HRCT-documented disease progression (Fig. 6A). A patient showed disease progression over time, HRCT of the chest demonstrated a progressive fibrotic pattern (Fig. 6B). During the follow-up, exosomal KL-6 levels rose from 161.07 U/mL to 331.2 U/mL, while plasma KL-6 levels increased from 296.48 U/mL to 838.72 U/mL. These biochemical changes were consistent with the progressive fibrotic alterations observed on HRCT.

## Discussion

This multi-center study provides compelling evidence for the superior diagnostic utility of exosomal KL-6 compared to its plasma counterpart in the context of CTD-ILD. Our key findings demonstrate that exosomal KL-6 significantly outperforms plasma KL-6, particularly in two critical clinical scenarios: (1) identifying CTD-ILD (plasma KL-6-) patients, and (2) distinguishing CTD-ILD (plasma KL-6-) patients from CTD (Non-ILD). Specifically, exosomal KL-6 achieved high diagnostic accuracy (AUC = 0.900) for differentiating CTD-ILD (plasma KL-6-) patients from HCs, significantly surpassing plasma KL-6 (AUC = 0.879). More importantly, for the clinically challenging differentiation of CTD-ILD (plasma KL-6-) from CTD (Non-ILD), exosomal KL-6 maintained good performance (AUC = 0.782, sensitivity 90.0%, specificity 77.3% at cutoff 120.87 U/mL), while plasma KL-6 showed a statistically significant but much weaker difference (*P* < 0.05) compared to exosomal KL-6 (*P* < 0.001), and yielded lower diagnostic power (AUC = 0.705).

The enhanced diagnostic performance of exosomal KL-6, especially in CTD-ILD (plasma KL-6-), can be plausibly explained by several biological advantages of exosomes. Firstly, exosomes are actively secreted vesicles reflecting the state of their parent cells. KL-6 packaged within exosomes likely originates more directly from injured or regenerating alveolar epithelial cells (AECs) in ILD, potentially offering a more disease-specific signal than plasma KL-6, which represents a passive leakage into the circulation and may be influenced by systemic inflammation or other sources [36, 37]. Secondly, the exosomal membrane may provide a protective environment that shields KL-6 from degradation by circulating proteases, potentially enhancing its stability compared to free plasma KL-6 [38]. Thirdly, exosomes derived from specific cell types are known to carry cell-specific protein cargoes; thus, exosomal KL-6 may originate more directly from injured alveolar epithelial cells, potentially offering a more disease-specific signal than plasma KL-6 [39]. This enrichment could explain why exosomal KL-6 remains detectable and diagnostically useful even when plasma levels fall below conventional positivity thresholds [40].

Although our single‑cell transcriptomic analysis (Fig. 2D–J) showed minimal MUC1 expression in mesenchymal cells (e.g., fibroblasts, myofibroblasts), we acknowledge the possibility of indirect mesenchymal–epithelial crosstalk. Activated fibroblasts in the fibrotic niche secrete pro‑fibrotic cytokines such as TGF‑β and IL‑6, which may upregulate MUC1 expression and promote exosomal KL‑6 release from AT2 cells. Thus, elevated exosomal KL‑6 could indirectly reflect the activity of the mesenchymal compartment and fibrotic progression. This notion is supported by our longitudinal data (Fig. 6), where exosomal KL‑6 levels increased in parallel with radiographic progression. Future studies using co‑culture systems or spatial transcriptomics are needed to directly test this hypothesis.

The finding of a remarkably high correlation between plasma and exosomal KL-6 concentrations confirms that these measures are closely related, likely reflecting the same underlying pathophysiological process of epithelial injury. However, their distinct diagnostic performances, particularly in differentiating CTD-ILD (plasma KL-6-) from CTD (Non-ILD), highlight a crucial nuance. While the concentrations move together, the biological context and stability provided by the exosomal compartment appear to confer a significant diagnostic advantage, especially at lower concentration ranges relevant to early or less severe disease. This suggests that exosomal KL-6 is not merely a surrogate for plasma KL-6 but may offer unique and clinically more relevant information.

Several limitations should be acknowledged. First, although our multi-center cohort provides robust internal validation, external validation in independent, diverse cohorts is necessary to confirm the generalizability of our findings. Second, some patients had initiated anti-inflammatory, immunosuppressive, or antifibrotic therapy prior to blood sampling. While such treatments may influence circulating KL-6 levels, KL-6 is primarily a marker of alveolar epithelial injury and fibrotic lung burden, which are unlikely to be substantially altered by short-term therapy. Consequently, we consider the impact of prior treatment on our overall findings to be limited; nevertheless, this potential bias cannot be entirely excluded. Future prospective studies with standardized pre- and post-treatment sampling are warranted to further validate our conclusions. Third, the longitudinal substudy included only 10 patients with radiographic progression and lacked a stable disease control group, limiting statistical power. Future larger longitudinal cohorts including both progressive and stable patients are needed to validate exosomal KL-6 as a progression biomarker. Finally, all participants were recruited from three centers in China. The diagnostic performance of exosomal KL-6 should be further evaluated in other ethnic populations and geographic regions.

## Conclusion

In summary, exosomal KL-6 is a significantly more accurate biomarker than plasma KL-6 for diagnosing CTD-ILD, particularly in CTD-ILD (plasma KL-6-). Its superiority likely arises from its direct biological origin and stability within exosomes. While highly promising for early detection, differentiation, and monitoring, validation in larger cohorts and further mechanistic research are crucial.

## Supplementary Information

Supplementary Material 1: Fig. s1 Characterization of exosomes and freeze-thaw stability. In this study, plasma-derived exosomes were characterized using NTA and TEM. NTA showed a median size of 61–62 nm, and TEM revealed typical cup-shaped vesicles with intact membranes and no debris, confirming high purity and integrity. Fresh (4°C) and once freeze-thawed (–80°C) samples from the same patients were compared. No significant differences were observed in exosomal KL-6 levels, particle size, or particle concentration (Supplementary Fig. 1A–C). TEM also confirmed unchanged morphology after freeze-thaw. These data indicate that a single freeze-thaw cycle does not cause significant exosome lysis or loss of integrity, and thus does not affect exosomal KL-6 measurement reliability. In summary, our exosome isolation protocol yields high-quality exosomes, and the retrospective use of frozen samples in the main study is methodologically sound.

## Data Availability

The data that support the findings of this study are not openly available due to reasons of sensitivity and are available from the corresponding author upon reasonable request.
