# Supplementary material for: Exosomal KL-6 surpasses total plasma KL-6 in the diagnosis of Interstitial Lung Disease (ILD)
Source: Respir Res. 2026 Jul 28;27:418. doi: 10.1186/s12931-026-03816-x (PMC13693089; doi:10.1186/s12931-026-03816-x)
Supplement: Supplementary file 1 [file 12931_2026_3816_MOESM1_ESM.docx]

**Supplementary Figure 1. Characterization of exosomes and freeze-thaw stability.**
(A) Exosomal KL‑6 levels in fresh (4°C) versus freeze‑thawed (–80°C, one cycle) samples. (B). Size distribution of exosomes measured by nanoparticle tracking analysis (NTA) before and after freeze‑thaw cycle. (C) Particle concentration of exosomes before and after freeze‑thaw. (D) Representative transmission electron microscopy (TEM) images of exosomes before (upper panel) and after (lower panel) a single freeze‑thaw cycle. Scale bars: 200 nm (left) and 50 nm (right).

**Methods:**

To verify the size distribution, morphology, and purity of isolated exosomes, nanoparticle tracking analysis (NTA) and transmission electron microscopy (TEM) were performed on exosome samples from 5 randomly selected ILD patients. NTA was conducted using a Malvern Panalytical NS 300 system (Malvern Panalytical Ltd., UK) at a dilution of 1:1000. TEM imaging was performed using a 120 kV transmission electron microscope (Thermo Fisher Scientific, USA) at a dilution of 1:100. For freeze-thaw stability assessment, fresh plasma samples were split into two aliquots: one was stored at 4°C overnight (fresh control), and the other was stored at -80°C overnight then rapidly thawed to room temperature (freeze-thaw group). Exosomes were isolated from both aliquots and analyzed for KL-6 concentration, particle size, and particle concentration. Detailed results are presented in Supplementary Fig. 1.

**Results**

As shown in Supplementary Fig. 1, NTA analysis revealed that isolated exosomes had a median diameter of 61.50 nm (IQR 58.35–70.65 nm) before freezing and 62.20 nm (IQR 61.00–63.40 nm) after one freeze-thaw cycle, both within the typical exosome size range (30–150 nm) (Supplementary Fig. 1B). TEM images confirmed the presence of intact spherical or cup-shaped vesicles with no visible cellular debris or protein aggregates (Supplementary Fig. 1D). No significant differences were observed between fresh and freeze-thawed samples in KL-6 concentration (603.3 vs 588.3 U/mL, *P*>0.05), particle size (61.50 vs 62.20 nm, *P*>0.05), or particle concentration (3.51×10⁸ vs 4.45×10⁸ particles/mL, *P*>0.05) (Supplementary Fig. 1A–C). These data confirm that a single freeze-thaw cycle does not compromise exosome integrity or KL-6 measurement reliability.

**Discussion**

In this study, plasma-derived exosomes were systematically characterized using NTA and TEM. NTA results showed a median exosome size of approximately 61–62 nm, and TEM images revealed typical spherical or cup-shaped vesicular morphology with intact membranes and no visible cellular debris or protein aggregates. These features confirm the high purity and integrity of the exosome preparations used in this study.

Furthermore, we directly compared fresh (4°C) and once freeze-thawed (–80°C) exosome samples from the same patients. As shown in Supplementary Fig. 1A–C, there were no significant differences in exosomal KL-6 levels, median particle size, or particle concentration between the two conditions. TEM images also confirmed that vesicle morphology and size remained consistent after a single freeze-thaw cycle. These data indicate that a single freeze-thaw cycle does not cause significant exosome lysis or loss of particle integrity, and therefore does not affect the reliability of exosomal KL-6 measurements.

In summary, this supplementary analysis validates that the exosome isolation protocol used in this study yields high-quality exosomes, and that the retrospective use of frozen samples in the main study is methodologically sound.
